# *Glycine tabacina* (Labill.) Benth. Ethanol Extract Attenuates LPS-Induced Neuroinflammation and Behavioral Deficits by Modulating TLR4/NF-κB/NLRP3 Signaling Pathway

**DOI:** 10.3390/ijms27146440

**Published:** 2026-07-20

**Authors:** Boyang Qiu, Yichen Zhuo, Zixin Teng, Wendi Yue, Yongkai Liang, Zhimin Miao, Yuxin Zhao, Xiaobing Cui, Yunzhen Gao, Lan Zhang, Zeen He, Pui Man Hoi, Chengwei He

**Affiliations:** 1 State Key Laboratory of Mechanism and Quality of Chinese Medicine, University of Macau, Macau SAR 999078, China; mc36210@connect.um.edu.mo (B.Q.); yc57555@um.edu.mo (Y.Z.); mc46301@um.edu.mo (Z.T.); wendyyue20010220@163.com (W.Y.); kyle.y.liang@gmail.com (Y.L.); mzmdsg@163.com (Z.M.); yc27506@um.edu.mo (Y.Z.); yc37512@um.edu.mo (X.C.); yc47532@um.edu.mo (Y.G.); yc47522@um.edu.mo (L.Z.); mc46247@um.edu.mo (Z.H.); 2 Institute of Chinese Medical Sciences, University of Macau, Macau SAR 999078, China; 3 Department of Pharmaceutical Science, Faculty of Health Sciences, University of Macau, Taipa, Macao SAR 999078, China

**Keywords:** neuroinflammation, microglia, *Glycine tabacina*, behavioral deficit

## Abstract

*Glycine tabacina* (Labill.) Benth is commonly known as “Yan-Dou” and is a folk medicinal herb in China used to alleviate rheumatism. Although its anti-inflammatory and antioxidant activities have been reported, its effects on microglia-driven neuroinflammation, neuronal protection, and neuroinflammation-associated behavioral impairment have not been investigated. An LPS-stimulated microglial activation model was established using BV2 cells to evaluate the anti-neuroinflammation activity of *Glycine tabacina* extract (GTE) and to explore its underlying mechanisms. Neuroprotective efficacy was assessed using a BV2–HT22 conditioned interaction model to determine whether GTE mitigates microglia-mediated neuronal injury. A zebrafish model was used to examine the effects of GTE on LPS-induced neuroinflammatory phenotypes and behavioral deficits. GTE significantly inhibited LPS-induced neuroinflammation in BV2 microglia. GTE also showed a strong neuroprotective effect by suppressing HT22 cell death in the BV2-HT22 conditioned interaction model. Mechanistic assays indicated that the neuroprotective effects of GTE were associated with the inhibition of TLR4/NF-κB/NLRP3 signaling cascade. In vivo studies showed that GTE mitigated LPS-stimulated neuroinflammatory phenotypes in both peripheral and central compartments and significantly improved behavioral performance in zebrafish. The results of this study demonstrate remarkable neuroprotective and anti-neuroinflammatory effects of GTE through regulation of the TLR4/NF-κB/NLRP3 signaling pathway and highlight its potential as a therapeutic candidate for neuroinflammation-related CNS disorders.

## 1. Introduction

The central nervous system (CNS) is under constant surveillance by resident microglia and circulating immune cells like macrophages, dendritic cells, and T cells, which are vigilant against harmful agents to maintain CNS homeostasis [1]. Neuroinflammation refers to an inflammatory response within the CNS triggered by diverse insults such as infection, toxins, ischemia, and traumatic injury [2]. The broad and expanding application of the concept of neuroinflammation is exemplified by its linkage to numerous conditions, spanning obesity [3], pain [4], epilepsy [5], depression [6], schizophrenia [7], Alzheimer’s disease [8], Parkinson’s disease [9], and amyotrophic lateral sclerosis [10]. However, as the field has matured, a more nuanced understanding has emerged: the term “neuroinflammation” must be applied precisely. Accurate attribution typically requires consideration of multiple hallmarks, including elevated inflammatory mediators, immune cell infiltration, microglial activation, and evidence of neurodegenerative tissue damage, rather than relying on a single readout [11].

CNS primarily consists of two distinct cell types: neurons and glial cells. Neuroglia includes astrocytes, oligodendrocytes, microglia, and ependymal cells [12]. Microglia constitute roughly 10–15% of the total glial population in the mature brain [13]. In response to injury or pathogenic cues, microglia can adopt heterogeneous activation states. Historically, these have been simplistically described as pro-inflammatory “M1-like” and immunoregulatory “M2-like” phenotypes, although such binary classification does not fully capture microglial complexity [14]. A range of stimuli, including lipopolysaccharide (LPS), interferon-gamma (IFN-γ), β-amyloid (Aβ), and α-synuclein, have been shown to activate microglia through classical pathways in both lab settings and living organisms [15]. When faced with these insults, microglia respond by releasing a surge of proinflammatory cytokines, including interleukin-1 beta (IL-1β), interleukin-6 (IL-6), and tumor necrosis factor-alpha (TNF-α). Toll-like receptor 4 (TLR4) is one of the most prominently expressed toll-like receptors in microglia [16]. Research has extensively documented that TLR4-mediated microglial activation significantly contributes to neurodegenerative diseases [17]. LPS, a well-characterized TLR4 ligand, has been widely used to study microglial activation mechanisms in neurodegeneration. Notably, microglia are among the most responsive CNS cell types to LPS stimulation [16]. Upon binding, LPS interacts with LPS-binding protein (LBP) and the glycosylphosphatidylinositol-anchored protein CD14, forming a complex with TLR4 [18]. A central pathway downstream of TLR4 is NF-κB, a transcriptional regulator that drives the expression of inflammatory genes and promotes the production of cytokines and inflammatory mediators, including TNF-α, IL-6, and nitric oxide (NO), ultimately contributing to neuronal injury [19]. In addition to transcriptional inflammatory programs, inflammasome activation provides a potent amplification mechanism. The NLRP3 (NOD-like receptor family pyrin domain containing 3) inflammasome is the most extensively studied inflammasome in the CNS. It consists of the NLRP3 sensor, the adaptor ASC (apoptosis-associated speck-like protein containing a CARD), and pro caspase-1. Upon activation, inflammasome assembly promotes caspase-1 maturation, which in turn processes pro-IL-1β and pro-IL-18 into their active secreted forms [20]. Accumulating evidence suggests crosstalk between TLR4/NF-κB priming and NLRP3 inflammasome activation in neurodegenerative and neuroinflammatory conditions, thereby intensifying cytokine production and amplifying neuronal damage [21].

*Glycine tabacina* (Labill.) Benth (*G. tabacina*), known as “Yan-Dou” in Chinese, is distributed across Southeast Asia as well as Oceania, and is abundant in Fujian and Taiwan. Traditionally, wild *G. tabacina* roots have been employed as an herbal remedy for hundreds of years, yet its bioactive constituents and pharmacological mechanisms remain incompletely characterized [22]. Our previous studies have reported the therapeutic activities of *G. tabacina* extracts (GTE) against collagen-induced rheumatoid arthritis by inhibiting oxidative stress and activation of NF-κB and MAPK pathways [23,24]. Of note, GTE exhibits anti-inflammatory efficacy without obvious toxicity [24,25,26]. Moreover, phytochemical investigations have identified 31 compounds from GTE, including eight new coumestans (glytabastans A–H), some of which show anti-inflammatory activity [25].

Zebrafish larvae have emerged as a powerful model organism for investigating human diseases, including cardiovascular and inflammatory research [27]. Neuroinflammation can be experimentally induced in zebrafish larvae using agents such as copper sulfate CuSO_4_, lipopolysaccharide (LPS), acrylamide, and rifampicin [28]. Among these, LPS immersion has become a well-established in vivo approach for studying inflammatory processes [29]. The availability of transgenic lines, such as *Tg(mpx:EGFP)*, further enhances this system by enabling direct visualization of neutrophil recruitment during inflammatory responses [30]. Beyond inflammation, zebrafish larvae are increasingly recognized as a valuable in vivo platform for behavioral research. Their rapid development, genetic tractability, and suitability for high-throughput screening make them a particularly effective platform for modeling neurodegenerative disorders and facilitating drug discovery [31,32,33,34,35].

In this study, we investigated whether GTE suppresses LPS-induced microglial activation and confers neuroprotection in the microglia–neuron interaction paradigm. We further assessed whether GTE ameliorates LPS-induced neuroinflammatory phenotypes and behavioral deficits in zebrafish. Our data demonstrate remarkable neuroprotective and anti-neuroinflammatory effects of GTE via inhibiting the activation of the TLR4/NF-κB/NLRP3 signaling pathway and suggest its potential application in the prevention and treatment of neuroinflammation-related CNS disorders.

## 2. Results

### 2.1. Establishment of Neuroinflammation Model in BV2 Cells

**Figure 1:**
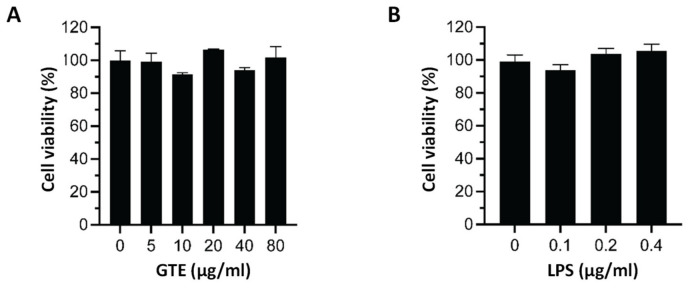

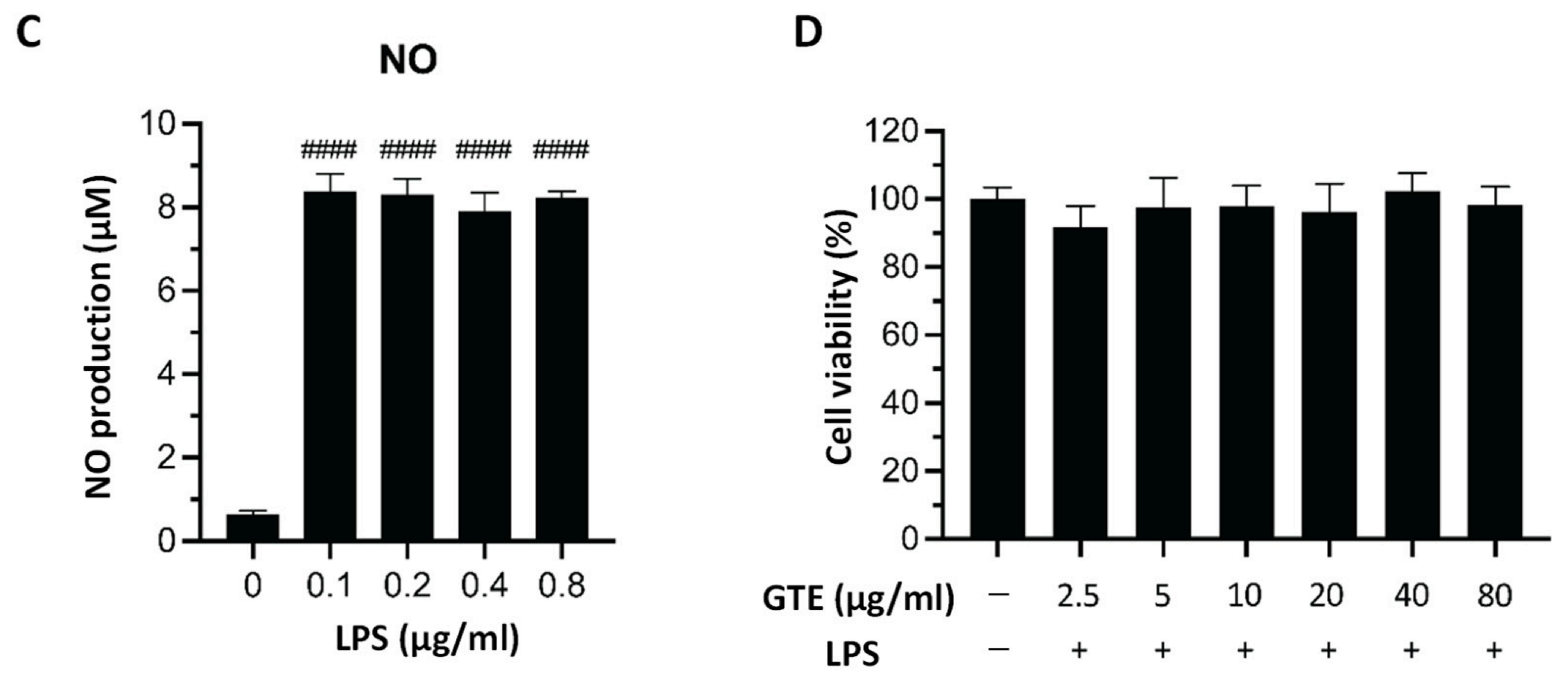
Establishment of cell inflammation model in BV2 cells. (**A**) Cells were incubated with *G. tabacina* extracts (GTE) (0, 5, 10, 20, 40, and 80 μg/mL) for 24 h. Cell viability was assessed using the CCK8 assay. (**B**) Cells were incubated with LPS (0, 0.1, 0.2, and 0.4 μg/mL) for 24 h. Cell viability was assessed using the CCK8 assay. (**C**) Cells were incubated with LPS (0.1 μg/mL) for 24 h. nitric oxide (NO) levels were measured using Griess kit. (**D**) Cells were co-treated with different concentrations of GTE and LPS (0.1 μg/mL) for 24 h. Data were expressed as means ± SD (*n* = 3). #### *p* < 0.0001 versus control group.

LPS-induced inflammatory response in BV2 cells was used to test the effect of GTE on neuroinflammation in vitro [16]. First, we used CCK8 assay to determine the safe concentrations of LPS and GTE for subsequent experiments. The results showed that GTE did no show obvious cytotoxicity at 80 μg/mL and below (Figure 1A), while LPS showed no cytotoxicity at concentrations up to 0.4 μg/mL (Figure 1B). NO production was applied to evaluate the inflammatory response in BV2 cells induced by LPS. The results showed the release of NO was significantly increased after LPS treatment (Figure 1C). Moreover, combined treatment of 20 μg/mL, 40 μg/mL, or 80 μg/mL GTE with 0.1 μg/mL LPS did not affect cell viability (Figure 1D). Then we chose these concentrations for the subsequent experiments.

### 2.2. GTE Inhibited LPS-Induced NO Release, Inflammatory Mediators’ Expression, and the Migratory Ability of BV2 Cells

**Figure 2:**
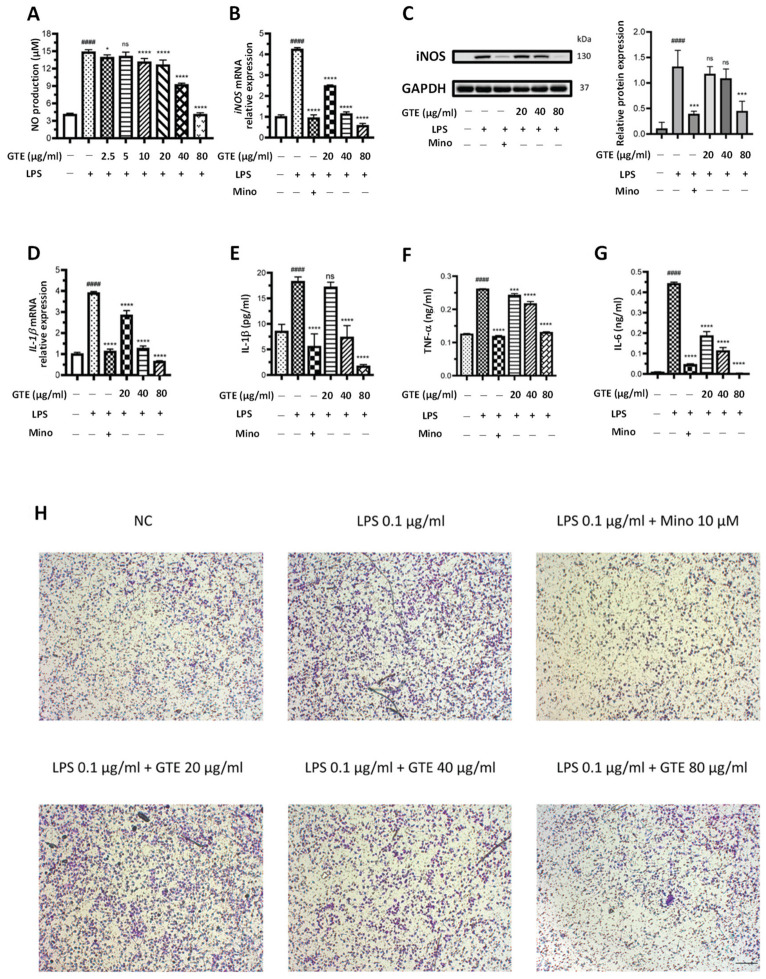
GTE inhibited LPS-induced NO release, expression of inflammatory cytokines and migratory potency of BV2 cells. Cells were stimulated with LPS (0.1 μg/mL) with or without different concentrations of GTE or 10 μM minocycline (positive drug) for 24 h. (**A**) NO levels were measured using Griess kits. (**B**) *iNOS* mRNA expression determined by RT-PCR. (**C**) iNOS protein expression determined by Western blot analysis. (**D**) *IL-1β* mRNA expression. Protein expression levels of IL-1β (**E**), TNF-α (**F**), and IL-6 (**G**) determined by ELISA kits. (**H**) Migration of BV2 cells determined by Transwell assay. Scale bar: 200 μm. Data are expressed as means ± SD (*n* = 3). #### *p* < 0.0001 versus control group; * *p* < 0.05, *** *p* < 0.001, **** *p* < 0.0001, and not significant (ns) versus LPS-treated group; Mino: minocycline (10 μM).

Activated microglia secrete a range of bioactive mediators—including cytokines, neurotransmitters, and toxic agents—that play key roles in triggering inflammatory responses [36]. The cells undergo significant shifts in gene expression and functionality, while the released bioactive mediators amplify the inflammatory response [19,37]. To evaluate the anti-inflammatory activity of GTE, expression of inflammatory factors was measured in a LPS-stimulated BV2 cell inflammation model. The results showed that NO production significantly increased after LPS stimulation, while GTE treatment significantly reduced the NO level (Figure 2A). Microglia do not express nitric oxide synthase (iNOS) at quiescent state, but express high levels of iNOS after stimulation for the synthesis of NO [38]. The results indicated that LPS remarkably induced the mRNA and protein expression of iNOS; however, GTE treatment significantly reduced the expression of iNOS in BV2 cells (Figure 2B,C). Furthermore, GTE treatment significantly inhibited the elevated expression of representative inflammatory factors, including IL-1β, TNF-α, and IL-6, induced by LPS in BV2 cells (Figure 2D–G). Microglia move toward and accumulate around pathogens or lesions to carry out inflammatory responses [39]. Activated glial cells secrete cytokines and chemokines that enhance inflammation by stimulating and recruiting additional cells [40]. The Transwell assay assessing cell migratory capacity revealed that LPS induced a heavy migration of BV2 cells. However, treatment with GTE markedly diminished the migration of BV2 cells stimulated by LPS (Figure 2H). These data demonstrate that GTE exhibits strong anti-neuroinflammatory activity in vitro.

### 2.3. GTE Suppressed the Activation of TLR4-NF-κB-NLRP3 Signaling Pathway in BV2 Cells

**Figure 3:**
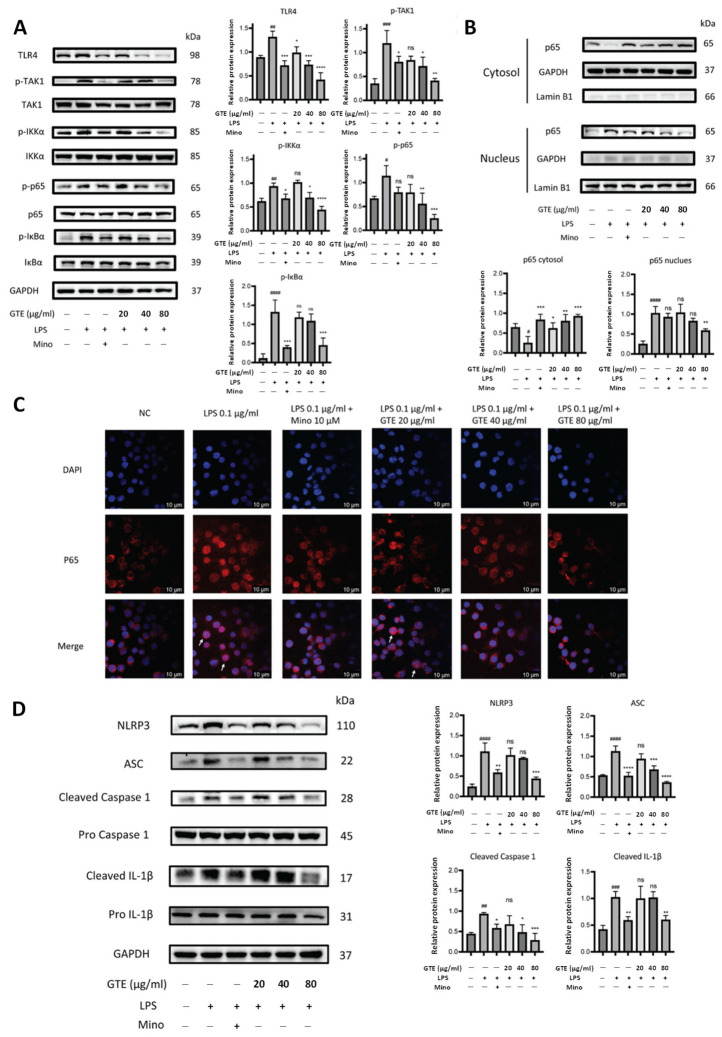
Effects of GTE on the activation of TLR4/NF-κB/NLRP3 pathway in LPS-stimulated BV2 microglia. BV2 cells were stimulated with LPS (0.1 μg/mL) with or without different concentrations of GTE or 10 μM minocycline (positive drug) for 3 to 24 h. (**A**) Protein expression levels were determined by Western blot. (**B**) p65 expression levels in nucleus and cytoplasm were determined by Western blot. GAPDH and Lamin B1 were used as internal loading controls. (**C**) I Immunofluorescence staining of p65 (red) was detected and visualized using a Single-photon Confocal Laser Scanning Microscope. Nuclei were stained with DAPI (blue). Scale bar: 10 μm. (**D**) Expression of NLRP3 inflammasome proteins was determined by Western blot. Data are expressed as means ± SD (*n* = 3). # *p* < 0.05, ## *p* < 0.01, ### *p* < 0.001, #### *p* < 0.0001 versus control group; * *p* < 0.05, ** *p* < 0.01, *** *p* < 0.001, **** *p* < 0.0001, and not significant (ns) versus LPS-treated group; Mino: minocycline (10 μM).

The transcription factor NF-κB plays a central role in driving inflammation. It directly triggers the expression of various pro-inflammatory genes and participates in the regulation of inflammasomes [41]. Western blot analysis was applied to assess the activation of TLR4/NF-κB pathway in BV2 cells. The LPS-treated group showed markedly higher levels of TLR4, p-TAK1, p-IKKα, p-p65, and p-IκBα compared to the control group, while GTE decreased the expression of these proteins compared with the LPS group (Figure 3A). These results indicate that GTE significantly inhibits the activation of the TLR4/NF-κB signaling pathway in BV2 cells induced by LPS. The translocation of p65 into the nucleus is a hallmark of cellular inflammation. Western blot analysis revealed that GTE treatment reduced the nuclear p65 levels, while increasing the cytoplasmic p65 protein level (Figure 3B), indicating GTE blocked p65 nuclear translocation and thereby attenuated inflammatory signaling. Moreover, the inhibitory effect of GTE on p65 nuclear translocation was also observed via immunofluorescence analysis (Figure 3C). These data demonstrate that the inhibition of inflammatory cytokine expression by GTE was attributed, at least partially, to the suppression of the TLR4/NF-κB signaling pathway.

The NLRP3 inflammasome, a member of the NOD-like receptor family characterized by its pyrin domain, stands as the most extensively researched inflammasome within the central nervous system. Research indicates that the TLR4/NF-κB pathway activates the NLRP3 inflammasome in neurodegenerative disorders [42]. To evaluate the effect of GTE on NLRP3 inflammasome, NLRP3-associated proteins were detected using Western blotting assay. GTE significantly decreased the expression of NLRP3, ASC, cleaved caspase-1, and cleaved IL-1β in BV2 cells stimulated by LPS (Figure 3D). These data indicated that the assembling of NLRP3 was suppressed by GTE treatment through inhibiting the TLR4/NF-κB signaling pathway, which further contributes to the suppression of inflammatory signaling.

### 2.4. GTE Inhibited HT22 Cell Apoptosis Triggered by Activated BV2 Cells

**Figure 4:**
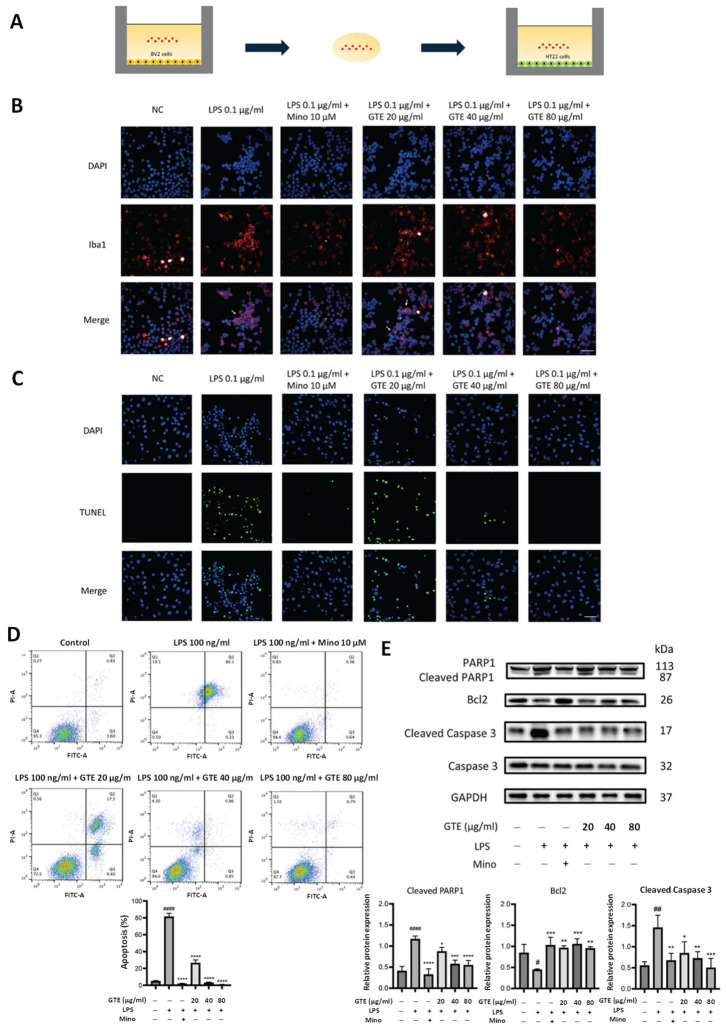
GTE suppressed HT22 cell apoptosis triggered by activated BV2 cells. (**A**) Schematic diagram shows the HT22 cell apoptosis model induced by conditional medium of activated BV2 cells. (**B**) Immunofluorescence staining of Iba1 (red, arrow) and nuclei staining with DAPI (blue). Scale bar: 100 μm. (**C**) Representative fluorescence images of TUNEL staining (green) in HT22 cells. Scale bar: 100 μm. (**D**) HT22 cell apoptosis analyzed by flow cytometry with Annexin V-FITC/PI staining. (**E**) Expression of apoptosis-related proteins in HT22 cells determined by Western blotting. Data are expressed as means ± SD (*n* = 3). # *p* < 0.05, ## *p* < 0.01, #### *p* < 0.0001 versus control group; * *p* < 0.05, ** *p* < 0.01, *** *p* < 0.001, **** *p* < 0.0001 versus LPS-treated group; Mino: minocycline (10 μM).

HT22 cells are widely used to study neuronal death mechanisms, including apoptosis [43]. To test the protective effect of GTE on neuronal apoptosis, we applied a HT22 cell apoptosis model induced by conditional medium of activated BV2 cells (Figure 4A). Iba1 (ionized calcium-binding adapter molecule 1) is specifically upregulated in microglia upon activation and is therefore used as a marker of activated microglia [40]. The results of immunofluorescence showed that LPS significantly induced the activation of BV2 cells, while GTE treatment robustly inhibited the activation of BV2 cells (Figure 4B). The results of TUNEL staining showed that LPS-treated BV2 cell conditional medium strongly induced HT22 cells apoptosis, while GTE treatment remarkably attenuated the apoptosis in HT22 cells (Figure 4C). In addition, Annexin V-FITC/PI assay demonstrated that the BV2 cell conditional medium caused 80% apoptosis in HT22 cells. However, treatment with GTE greatly diminished the apoptosis in a dose-depend manner (Figure 4D). Western blotting indicated that cleaved PARP1 and cleaved caspase-3 were significantly increased and Bcl2 was reduced in the conditional medium group, while GTE markedly reversed these effects (Figure 4E), further confirmed the anti-apoptotic activity of GTE. These data indicated that GTE treatment could significantly inhibit neuronal apoptosis induced by neuroinflammation in vitro.

### 2.5. GTE Alleviated LPS-Induced Inflammatory Responses in Zebrafish Larvae

**Figure 5:**
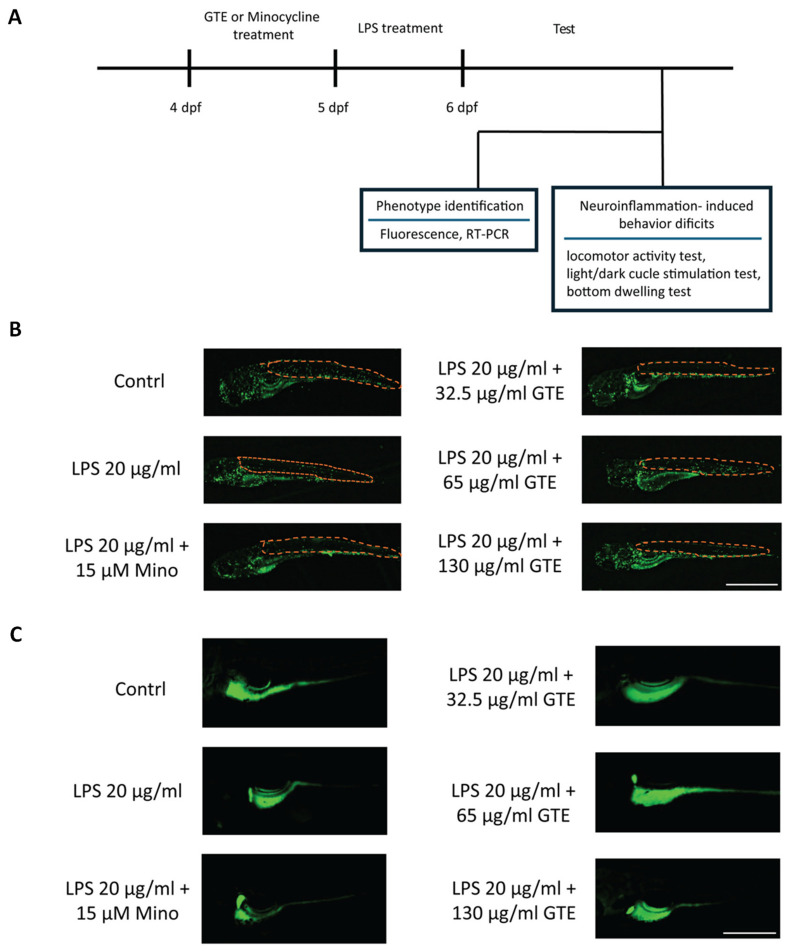

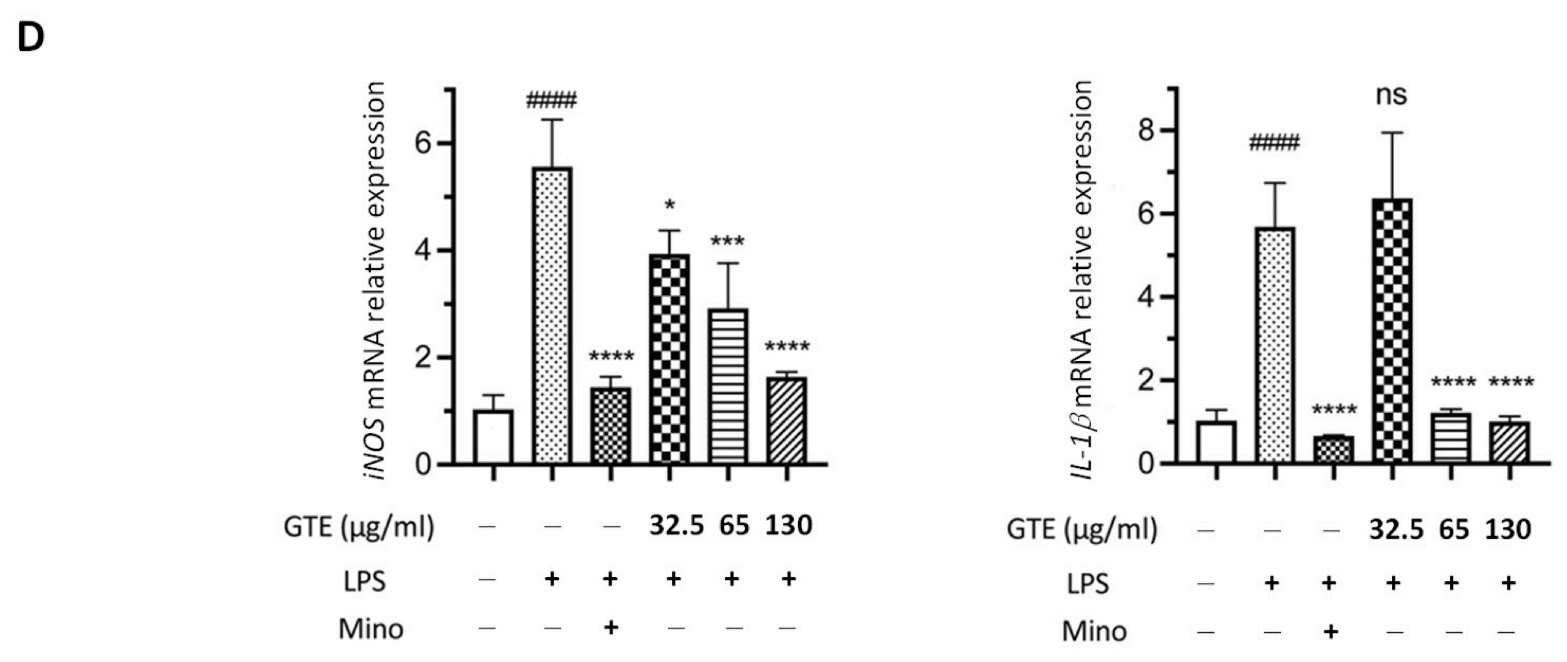
GTE attenuated LPS-induced inflammatory responses in zebrafish larvae. (**A**) Schematic illustration of the experimental design. (**B**) Representative images of neutrophil recruitment (area inside the orange dashed line) in transgenic zebrafish *Tg(mpx:EGFP)* in control and treatment groups. Scale bar: 100 μm. (**C**) The level of nitric oxide (NO) was measured by image analysis and fluorescence microscopy after staining with DAF-FM DA in AB zebrafish larvae. Scale bar: 100 μm. (**D**) mRNA expression levels of *iNOS* and *IL-1β* were evaluated by RT-PCR in AB zebrafish larvae. Data are presented as means ± SD (*n* = 6) analyzed by one-way ANOVA followed by Tukey’s post hoc test. #### *p* < 0.0001 versus control group; * *p* < 0.05, *** *p* < 0.001, **** *p* < 0.0001, and not significant (ns) versus LPS-treated group; Mino: minocycline (15 μM).

In this study, zebrafish larvae at 4 days post-fertilization (dpf) were pretreated with GTE or minocycline for 24 h, followed by exposure to LPS added to the medium for an additional 24 h (Figure 5A). *Tg(mpx:EGFP)* zebrafish larvae exposed to LPS alone led to a significant increase in neutrophil recruitment to the trunk region near the lateral line compared to control. Pretreatment with GTE at concentrations of 32.5, 65, and 130 μg/mL dose-dependently attenuated neutrophil recruitment relative to the LPS-only group (Figure 5B). The anti-inflammatory effect of GTE was comparable to that of minocycline (15 μM) (positive control). Furthermore, nitric oxide (NO) levels in zebrafish larvae (AB strain), measured using the fluorescent probe 4-amino-5-methylamino-2′,7′-difluorofluorescein diacetate (DAF-FM DA), were significantly elevated following LPS exposure, and this increase was markedly inhibited by GTE pretreatment (Figure 5C). Consistent with these findings, mRNA expression levels of *iNOS* and *IL-1β* were significantly reduced by GTE in LPS-treated larvae (Figure 5D). Collectively, these results demonstrate that GTE effectively mitigates LPS-induced inflammatory responses in zebrafish larvae in vivo.

### 2.6. GTE Alleviated Neuroinflammation-Caused Behavioral Deficits in LPS Induced Zebrafish Larvae

**Figure 6:**
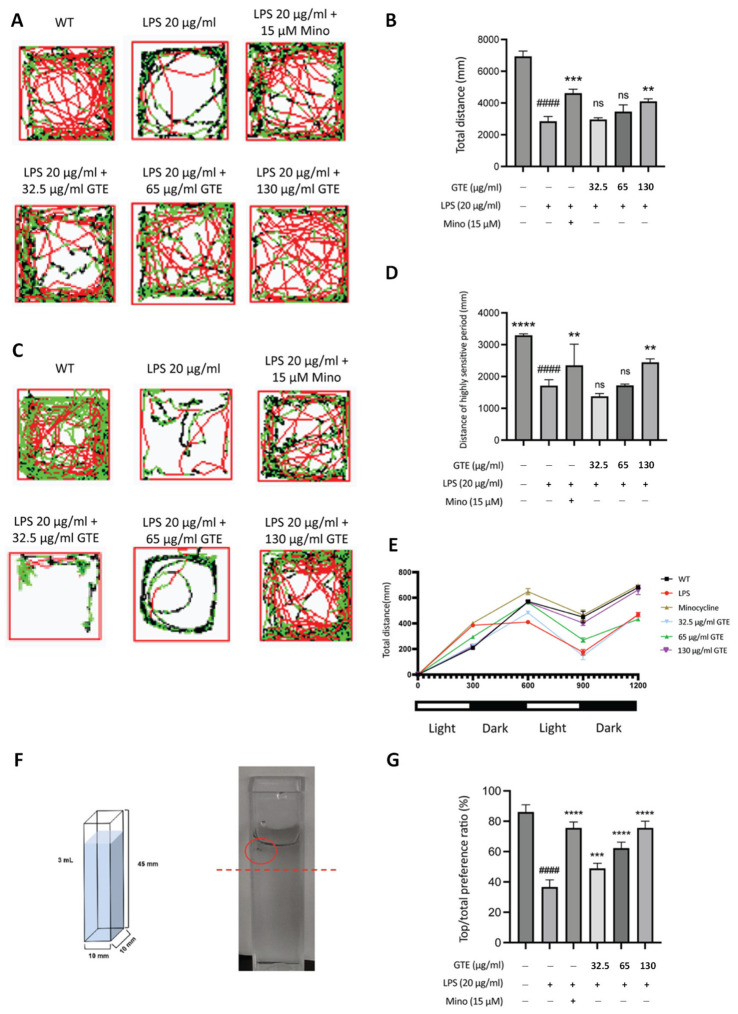
GTE alleviated behavioral impairments induced by LPS-mediated neuroinflammation in zebrafish larvae. (**A**) Representative locomotor patterns during the dark phase. Red line: fast motion trajectory (highly sensitive phase). Green line: medium-speed motion trajectory. Black line: slow/stationary trajectory. (**B**) Total distance traveled over a 5 min dark phase in (**A**). (**C**) Representative trajectories during a 20 min light/dark cycle. (**D**) Distance traveled during the highly sensitive phase of the light/dark cycle in (**C**). (**E**) Total distance covered across the 20 min light–dark cycle in (**C**). (**F**) Experimental setup for the bottom-dwelling assay chamber; larval position (as in the red circle) was recorded every 10 s, and the ratio of time spent in the upper zone (above the red dashed line) relative to total time was calculated. (**G**) Quantitative analysis of the ratio of upper zone to total during the bottom-dwelling assay. Data are expressed as means ± SD (*n* = 3). #### *p* < 0.0001 versus control group; ** *p* < 0.01, *** *p* < 0.001, **** *p* < 0.0001, and not significant (ns) versus LPS-treated group; Mino: minocycline (15 μM).

Zebrafish larvae have become an increasingly valuable in vivo model for behavioral research, offering a rapid and effective system for studying neurodegenerative disorders and drug discovery [31,32,33,34,35]. Behavioral outcomes in zebrafish larvae exposed to LPS were assessed using a locomotor activity test, light/dark cycle stimulation test, and bottom-dwelling test. Treatments with GTE and minocycline were evaluated for their potential to mitigate LPS-induced behavioral impairments. LPS exposure significantly reduced travel distance compared with control, while both GTE- and minocycline-treated groups showed a significant improvement in locomotor activity (Figure 6A,B). All groups exhibited typical light/dark sensitivity, with reduced movement in light and increased speed in darkness. LPS-treated group demonstrated a significant decline in distance, while GTE- and minocycline-treated groups displayed marked increase relative to the model group. (Figure 6C–E). In the bottom dwelling test, zebrafish larvae were placed in standard cuvettes following treatment (Figure 6F). Exposure to LPS significantly reduced the initial exploratory activity of zebrafish larvae during the 5 min novel tank diving assay, indicative of heightened anxiety-like behavior. Treatment with either minocycline or GTE effectively reversed the behavioral alterations induced by LPS, indicating a reduction in anxiety-like responses (Figure 6G). Taken together, the behavioral analyses consistently demonstrate that GTE mitigates neuroinflammation-associated deficits triggered by LPS exposure.

## 3. Discussion

Neuroinflammation refers to the inflammation in the nervous system in response to various harmful stimuli, such as infections, injuries, toxins, and autoimmune processes. Sustained and uncontrolled neuroinflammation is implicated in the pathogenesis of multiple CNS disorders, such as stroke, multiple sclerosis, Alzheimer’s disease (AD), and Parkinson’s disease (PD) [44]. Microglia constitute the inherent immune population in CNS, functioning as the initial line of defense against diverse pathological conditions [13]. Evidence supports that excessive microglial activation contributes to neuronal injury and cognitive dysfunction, particularly in vulnerable regions such as the hippocampus [15,19,45]. Thus, drugs which can restore microglial balance may offer therapeutic potential for neuroinflammation.

In this study, we evaluated the anti-neuroinflammatory and neuroprotective activities of the *Glycine tabcina* extract (GTE) using both in vitro microglial activation paradigms and in vivo zebrafish model. In BV2 microglia stimulated with LPS, GTE significantly reduced the production of inflammatory mediators, including NO and proinflammatory cytokines (TNF-α, IL-6, and IL-1β). Immunofluorescence staining showed that GTE robustly inhibited the LPS-induced overexpression of Iba1 (a hallmark of microglial activation) in BV2 cells. In addition, GTE attenuated microglial migration ability in the chemotaxis assay, suggesting that GTE suppresses microglial mobility which accompanies inflammatory activation. Together, these data indicate that GTE effectively suppresses LPS-induced microglial inflammatory phenotypes.

TLR4 is a principal pattern-recognition receptor mediating LPS-driven activation of microglia and has been implicated in neuroinflammation and neurodegenerative progression [17]. The canonical NF-κB activation involves IκBα phosphorylation and degradation, leading to nuclear translocation of NF-κB and transcriptionally upregulated expression of inflammatory cytokine [46] and NLRP3 inflammasome components [47]. Consequently, it triggers and amplifies inflammatory responses. Our results demonstrated that GTE directly suppressed upstream signaling by reducing TLR4 protein expression. Mechanistically, GTE prevented the phosphorylation of TAK1 (p-TAK1) and IKKα/β (p-IKKα), thereby inhibiting the phosphorylation and subsequent degradation of IκBα, and consequently prevented the release and nuclear translocation of the p65 NF-κB subunit. We confirmed this by showing that GTE reduced nuclear p65 levels while increasing cytoplasmic p65, as evidenced by both Western blotting and immunofluorescence. As a consequence, GTE significantly inhibited the expression of downstream pro-inflammatory cytokines, including TNF-α, IL-6, and IL-1β, as well as expression of iNOS and production of NO, a key inflammatory mediator, strongly confirming the anti-neuroinflammatory activity of GTE and explaining the underlying mechanisms.

Beyond the transcriptional regulation in inflammatory responses, inflammasome activation represents an important amplification mechanism in neuroinflammation. NLRP3 is a key pattern recognition receptor. The components of NLRP3 inflammasome, including NLRP3, ASC, and caspase-1, are expressed in microglia and astrocytes and drive the caspase-1 activation and maturation of IL-1β, thereby amplifying inflammatory responses [42,48]. NLRP3 inflammasome can induce chronic inflammation which may trigger an uncontrollable release of cytokines, ultimately causing neuronal death and neurodegenerative diseases. In this study, the LPS-induced activation of NLRP3 inflammasome was significantly suppressed by GTE, as evidenced by the downregulation of NLRP3, ASC, and caspase-1 in BV2 cells treated with GTE. Since NF-κB serves as a transcriptional primer for NLRP3 inflammasome components (NLRP3, pro-IL-1β, and pro-caspase-1), GTE-mediated inhibition of NF-κB leads to decreased expression of NLRP3 and ASC. Consequently, the assembly of the inflammasome complex is disrupted, resulting in reduced cleavage of pro-caspase-1 into its active form (cleaved caspase-1) and diminished maturation of IL-1β (cleaved IL-1β). Thus, GTE effectively acts as a “double brake” on the inflammatory cascade: it halts the transcriptional wave of pro-inflammatory genes via NF-κB while simultaneously disarming the inflammasome-mediated amplification and maturation of IL-1β. This interconnected inhibition is central to its potent anti-neuroinflammatory activity.

LPS-activated BV2 microglia release a neurotoxic cocktail containing high levels of TNF-α, IL-6, IL-1β, and NO. Co-treatment with GTE during LPS stimulation fundamentally altered the inflammatory secretome of BV2 cells by suppressing the TLR4/NF-κB/NLRP3 cascade. This GTE-modified conditioned medium, when transferred to HT22 neurons, exhibited significantly reduced neurotoxic potential, as evidenced by a marked reduction in TUNEL-positive apoptotic HT22 neurons; a dose-dependent decrease in the percentage of Annexin V-FITC/PI-positive apoptotic cells by flow cytometry; and a reversal of apoptotic protein markers in HT22 cells, including downregulation of cleaved PARP1 and cleaved caspase-3, and restoration of the anti-apoptotic protein Bcl. Importantly, we confirmed that GTE directly inhibits BV2 microglial activation (as shown by reduced Iba1 fluorescence), which is the primary source of the toxic mediators. Therefore, GTE’s neuroprotection is predominantly achieved through a phenotypic re-education of microglia—shifting them from a neurotoxic, pro-inflammatory state toward a less inflammatory, neuroprotective state, which subsequently spares HT22 neurons from microglia-mediated apoptotic injury.

Zebrafish are widely used as an in vivo model for investigating toxicity and inflammation. Their innate immune system, including neutrophil and macrophage phagocytic functions and conserved inflammatory pathways, closely parallels that of humans, making them suitable for studies of neuroinflammation and neurodegeneration [27,36]. Neuroinflammation was effectively induced in zebrafish through LPS stimulation, while GTE significantly reduced LPS-induced neutrophil recruitment to the trunk region near the lateral line and decreased NO-related signals in the head region of in transgenic larvae, indicating an anti-neuroinflammatory activity of GTE. Zebrafish larvae are well-established model in behavioral neuroscience due to their rapid development, genetic accessibility, and transparent physiology, which facilitates in vivo observation of neural activity. By 6 days post-fertilization (dpf), they exhibit a wide range of quantifiable behaviors, including locomotor activity, startle responses, and light/dark preference [49]. Our data demonstrated that GTE ameliorated LPS-associated behavioral deficits, including impaired locomotor activity, response to light/dark transitions, and bottom-dwelling behavior, further reinforces the anti-neuroinflammatory effect of GTE. Because behavioral outputs integrate multiple physiological systems, future work should determine whether the behavioral improvement is primarily attributable to the suppression of inflammation and neuronal apoptosis. Additionally, the underlying molecular mechanisms in vivo warrant further elucidation.

For in vitro cellular assays, GTE was applied at concentrations up to 80 μg/mL, which are non-cytotoxic to the tested cell lines. For in vivo studies in zebrafish larvae, the maximum GTE concentration was set at 130 μg/mL, as no overt toxicity was observed at or below this level. We note that these two concentration ranges are not directly comparable: in cell culture, GTE is taken up directly by cells in the medium, whereas GTE absorption in live zebrafish involves complex in vivo pharmacokinetic processes. This difference accounts for the relatively higher concentration used in in vivo experiments. Given the absence of published data on the blood–brain barrier (BBB) permeability of GTE’s major active components, we conducted in silico prediction using two publicly accessible online tools (https://nova.disfarm.unimi.it/vegaol/bbbp.htm (accessed on 5 July 2026) and http://bbbper.mdu.ac.in (accessed on 5 July 2026)). The results show that the principal active constituents of GTE—including glytabastan B, glytabastan D, coumestrol, and daidzein—are BBB-permeable. This finding further supports the observed protective effects of GTE against neuroinflammation and inflammation-induced behavioral deficits in zebrafish.

Overall, our results suggest that GTE suppresses microglial inflammatory activation and confers neuroprotection, at least in part through modulation of the TLR4/NF-κB/NLRP3 signaling network. These findings support the continued investigation of GTE and its active constituents as potential therapeutic candidates for neuroinflammation-related CNS disorders.

## 4. Materials and Methods

### 4.1. Antibodies and Reagents

The antibodies and regents used in our experiments are listed in Table 1.

### 4.2. Cell Culture and GTE Preparation

The immortalized mouse microglial BV2 and hippocampal neuronal HT22 cell lines were purchased from Pricella Biotechnology Co., Ltd. (Wuhan, China). Specific mediums and defined conditions were used to culture these cell lines. Both cell lines were cultured in Dulbecco’s modified Eagle’s medium (DMEM), supplemented with 10% Fetal Bovine Serum (FBS) and 1% penicillin–streptomycin, and incubated in a humidified incubator (Thermo Scientific, Carlsbad, CA, USA) at 37 °C in 5% CO_2_:95% air. These cell lines have been extensively used for pre-clinical studies of CNS diseases.

The ethanol extract of *G. tabacina* (GTE) was prepared, and its active chemical components were identified as described in our previous reports [22,23,25]. The chromatographic fingerprint of GTE was determined by Ultra Performance Liquid Chromatography (UPLC) on ACQUITY HCLASS PLUS UPLCTM coupled with a photodiode array (PDA) detector. Data were acquired and analyzed using Empower software version 3. The quantitative analysis of GTE was performed using the same UPLC system and column, as well as the same analytical software. The fingerprint of the overall chemical composition of GTE is shown in Figure S1. The contents of representative active compounds, including glytabastan B, glytabastan D, coumestrol, and daidzein, in GTE were 0.0171 ± 0.0105%, 0.0020 ± 0.0011%, 0.0266 ± 0.0054%, and 0.6685 ± 0.2554%, respectively (Table S1). The GTE was dissolved in purified water (ddH_2_O) via ultrasonic treatment. Minocycline hydrochloride, purchased from Rhawn company (Shanghai, China), was dissolved in DMSO and sonicated. LPS, purchased from Sigma-Aldric (St. Louis, MO, USA), was dissolved in phosphate-buffered saline (PBS).

### 4.3. Cell Viability

CCK8 assay was performed to determine the cell viability. Initially, cells were seeded in 96-well plates; each well contained 1 × 10^4^ cells with 100 µL of DMEM complete medium and was cultured for 24 h. The cells were treated with different dosages of LPS or GTE for 24 h, following by replacing the medium with 100 μL of DMEM containing 10% CCK8 solution per well, and incubating for another 1.5–2 h in dark. Subsequently, the optical density (O.D.) value was measured by a microplate reader (PerkinElmer, Waltham, MA, USA) at 450 nm with reference wavelength of 650 nm. The OD values were recorded, and cell viability was calculated by following equation:

$$\mathrm{Relative}\mathrm{Cell}\mathrm{Viability}(\%)=\frac{O.D.\mathrm{of}\mathrm{treatment}-O.D.\mathrm{of}\mathrm{treatment}\mathrm{blank}}{O.D.\mathrm{of}\mathrm{control}-O.D.\mathrm{of}\mathrm{control}\mathrm{blank}}\times 100$$

### 4.4. GTE Treatment and Model Establishment

Three concentrations (20 μg/mL, 40 μg/mL, 80 μg/mL) of GTE were used in in vitro experiments. The concentration of LPS was determined by the rate of nitric oxide (NO) inhibition which was selected as the criterion for modeling evaluation. The NO (Griess) kit purchased from Beyotime (Shanghai, China) is a simple and quick reagent for nitric oxide detection. BV2 cells were seeded in 96-well plates; each well contained 3 × 10^4^ cells with 100 µL of DMEM complete medium, and was cultured for 24 h, followed by the treatment of LPS for another 24 h. The cell supernatant was extracted, and NO quantification was performed using a NO detection kit (Beyotime, Shanghai, China) following the manufacturer’s protocol. According to the rate of NO inhibition, the stimulatory concentration was determined to be 0.1 μg/mL.

The detection of nitric oxide within zebrafish embryos was performed using the DAF-FM DA fluorescent probe (Biosharp). Essentially, DAF-FM DA (10 μM) was introduced into the zebrafish larvae after stimulation and treatment, then subjected to a 30 min dark incubation at 28.5 °C. Subsequently, the probe was rinsed out thrice with medium. Larval images were conducted using a Confocal Imaging System from the Disk Scanning Unit (DSU) Confocal Imaging System (Olympus Co., Tokyo, Japan).

### 4.5. RNA Isolation and Reverse Transcription-Polymerase Chain Reaction (RT-PCR)

Samples of 1 × 10^6^ cells were seeded in 6-well plates with LPS stimulation and GTE treatment for 24 h. Total RNA was extracted using M5 HiPer Total RNA Extraction Reagent (Mei5 Biotech, Beijing, China) according to the manufacturer’s protocol. The concentration and purity of RNA were determined on NanoDrop (ThermoFisher Scientific, Waltham, MA, USA). cDNA was synthesized using a reverse transcription kit (TAKARA, Japan) and a PCR c1000 thermal cycler (BioRad, Hercules, CA, USA). The cDNA was mixed with corresponding primers (IGE Biotechnology, Guangzhou, China) and SYBR Green PCR MasterMix (ThermoFisher, USA). RT-PCR was performed on QuantStudio 7 Flex Real-Time PCR System (ThermoFisher, USA). The primer sequences of each gene are listed in Table 2.

For the zebrafish specimens, groups of thirty larvae were anesthetized with 0.02% tricaine. The head regions, omitting the eyes and yolk sac, were then dissected out. All subsequent procedures followed the same protocol outlined previously. The sequences of primers used for each gene are provided in Table 3.

### 4.6. Cytokine Release Determination

Samples of 1 × 10^5^ cells were seeded in 24-well plates with LPS stimulation and GTE treatment for 24 h. Cell supernatant was collected. TNF-α, IL-6, and IL-1β were determined by the corresponding ELISA kits (Biolegend, San Diego, CA, USA) following the manufacturer’s protocol.

### 4.7. Protein Preparation and Western Blot Analysis

Samples of 1 × 10^6^ cells were seeded in 60 mm dishes with LPS stimulation and GTE treatment for 24 h. Protein was extracted using RIPA buffer with 1% of cocktail and 10% of PMSF (Beyotime, China). Protein concentration was measured using a BCA assay kit (ThermoFisher, Waltham, MA, USA). Protein samples were mixed with loading buffer (Beyotime, China) and denatured at 100 °C for 10 min with shaking at 650 rpm. Protein samples were separated on SDS-PAGE and transferred onto a PVDF membrane. The membrane was then blocked with 2.5% non-fat milk powder (Biorad, USA) for 2 h at room temperature. The membrane was incubated with primary antibodies (diluted 1:1000) at 4 °C overnight and then with a secondary antibody (diluted 1:2000, sourced from Beyotime) for 1.5 h at room temperature. The Ultra High Sensitivity ECL Kit (MCE, Monmouth Junction, NJ, USA) was used to develop and visualize the protein bands. Images were captured using ChemiDoc systems (Biorad, USA) in chemiluminescent and colorimetric settings. The density of the bands was quantified using ImageJ software version 1.51.

### 4.8. Transwell Assay

Samples of 1 × 10^5^ cells were seeded in 24-well plates with LPS stimulation and GTE treatment. Samples of 6 × 10^4^ cells were seeded with 200 μL penicillin–streptomycin-free DMEM complete medium in the upper chamber and then put in the wells of a Transwell plate to incubate for 24 h. Following incubation, the cells remaining in the upper chamber were carefully aspirated, while those that had successfully migrated through the membrane were fixed using a 4% paraformaldehyde solution for 15 min. The invasive cells were then stained with crystal violet for 15 min. The membrane was washed 3 times with PBS before being left to air-dry overnight. Finally, microscopic images were captured using cross-polarized light filters to enhance visualization.

### 4.9. Immunofluorescence

Samples of 1 × 10^4^ cells were seeded in 96-well plates with LPS stimulation and GTE treatment for 24 h. The cells were fixed by 4% paraformaldehyde for 10 min. Then the cells were treated with 100 μL of PBS containing 0.1% Triton X-100 and 1% BSA for 1 h. Next, primary antibodies diluted to the recommended concentration were added and incubated overnight at 4 °C. The cells were then rinsed 3 times with PBS. Subsequently, a secondary antibody was applied and allowed to react for 1 h at room temperature in the dark. After that, an anti-fluorescence quenching mounting solution containing DAPI (Beyotime, China) was added on the cells. Images were captured with Opera Phenix High Content Screening System or Single-photon Confocal Laser Scanning Microscope.

### 4.10. BV2-HT22 Conditional Medium Model

Samples of 1 × 10^6^ or 3 × 10^4^ BV2 cells were seeded in 6-well plate or 96-well plate with LPS stimulation and GTE treatment. HT22 cells were seeded in another 6-well plate with 1 × 10^6^ cells each well or 96-well plate with 1 × 10^4^ cells each well for 24 h. After stimulation and treatment, supernatants of BV2 were collected and transferred into the wells containing HT22 cells after their medium was discarded in advance. The treated HT22 cells were used in the subsequent experiments.

### 4.11. TUNEL Assay

Apoptosis in HT22 cells was determined using an EZ 488 TUNEL apoptosis detection kit (Life-iLab, Shanghai, China) according to the manufacturer’s protocol. The apoptosis index was calculated as follows:

$$\mathrm{Apoptosis}\mathrm{index}(\%)=\frac{\mathrm{apoptotic}\mathrm{cells}}{\mathrm{total}\mathrm{cells}}\times 100$$

### 4.12. Flow Cytometry Assay

Flow cytometry is utilized to determine the apoptosis in HT22 cells, which was further determined using Annexin V-FITC/PI double staining kit (Beyotime, Shanghai, China) by flow cytometry according to the manufacturer’s protocol. Data were analyzed using FlowJo software version 10.4.6.

### 4.13. Zebrafish Maintenance and Treatment

AB wild-type and the transgenic line Tg(Mpo:EGFP) zebrafish were obtained from the Animal Research Center at the University of Macau. The zebrafish were housed according to standard protocols outlined in the Zebrafish Handbook [50]. Adults were kept at 28.5 °C with a controlled 14 h light/10 h dark cycle. Embryos were cultured in a specialized embryo medium (0.54 mM KCl, 13.7 mM NaCl, 0.044 mM KH_2_PO_4_, 0.025 mM Na_2_HPO_4_, 0.1 mM MgSO_4_, 0.13 mM CaCl_2_, and 42 μM NaHCO_3_; pH 7.4) at a temperature of 28.5 °C.

The 4-day post-fertilization (4-dpf) zebrafish larvae were divided equally into different groups in a 6-well plate and exposed in medium containing LPS (20 μg/mL) and different concentrations of GTE for 24 h. Caudal hematopoietic tissue (CHT) and neutrophils at lateral line neural crest region were observed using a Disk Scanning Unit (DSU) Confocal Imaging System (Olympus Co., Tokyo, Japan) to assess the efficacy of GTE in mitigating neuroinflammation.

### 4.14. Zebrafish Motor Behavior Evaluation

Following LPS stimulation and GTE treatment, 6-dpf zebrafish larvae were placed into a 96-well plate (1 fish/well). Locomotion of zebrafish was tracked for 30 min under darkness via a zebrafish tracking system (Viewpoint Life Sciences, Lyon, France). The cumulative movement distance of zebrafish larvae was measured concurrently.

Additionally, two behavioral experiments were conducted: a light–dark cycle assessment and a bottom-dwelling observation. For the light–dark cycle test, zebrafish were given a 10 min acclimation period before undergoing alternating 5 min light and dark phases, separated by 5 min intervals. This sequence was cycled twice. In the bottom-dwelling experiment, larvae were individually placed in Fisherbrand standard cuvettes (10 mm × 10 mm × 45 mm, Cat. No. 14955130) containing 3 mL of E3 medium. As for the bottom-dwelling test, the cuvettes were partitioned into top and bottom sections, and after a 10 min habituation period, the larvae’s position was tracked at 10 s intervals for 5 min. The bottom-dwelling tendency was quantified by calculating the proportion of time spent in the top zone relative to the total observation period.

### 4.15. Statistical Analysis

Statistical analyses were conducted utilizing GraphPad Prism 8.0 (San Diego, CA, USA). Each experiment was performed at least 3 times, and the results were presented as mean ± SD. Shapiro–Wilk Test was utilized to confirm all the numeric data were consistent with a normal distribution. One-way Analysis of Variance (ANOVA) with Tukey’s multiple comparison test were performed to evaluate the statistically significant variances among multiple groups. Values of *p* less than 0.05 were defined as significant.

## 5. Conclusions

In summary, this study investigated the efficacy and underlying molecular mechanisms of GTE in alleviating neuroinflammation in vitro and in vivo. In LPS-stimulated BV2 microglia, GTE reduced the production of proinflammatory factors (NO, TNF-α, IL-6, and IL-1β), which is in line with the suppression of the TLR4/NF-κB/NLRP3 signaling axis. In a BV2-conditional medium-induced HT22 cell apoptosis model, GTE remarkably enhanced neuronal survival, supporting that attenuating microglial inflammatory outputs can mitigate microglia-associated neuronal injury. In zebrafish larvae in vivo, GTE attenuated LPS-induced inflammatory phenotypes in peripheral and central compartments and significantly improved behavioral performance in locomotor, light/dark, and bottom-dwelling assays. Collectively, these findings provide mechanistic and functional evidence supporting GTE as a promising candidate remedy for the treatment of neuroinflammation-associated CNS disorders.

## Figures and Tables

**Table 1:** The antibodies and reagents for this study.

Cat. No	Items	Manufacturers
12800082	Dulbecco’s modified eagle medium (DMEM)	Gibco, Grand Island, NY, USA
003002	Phosphate-Buffered Saline (PBS)	Gibco
10270106	Fetal Bovine Serum (FBS)	Gibco
25200056	Trypsin-EDTA (0.05%), phenol red	Gibco
C0222	Penicillin-Streptomycin Solution, 100X	Beyotime, Shanghai, China
L4391	Lipopolysaccharides from *Escherichia coli* 0111: B4	Sigma-Aldric, St. Louis, MO, USA
R013129	Minocycline hydrochloride	Rhawn, Shanghai, China
D8371	Dimethyl sulfoxide (DMSO)	Solarbio, Beijing, China
C0038	CCK-8 kit	Beyotime
S0021M	NO (Griess) kit	Beyotime
MF034-01	M5 HiPer Total RNA Extraction Reagent	Mei5bio, Beijing, China
RR037A	PrimeScript^TM^ RT reagent Kit (Perfect Real Time)	Takara, Kusatsu, Shiga, Japan
2764334	PowerUp^TM^ SYBR^TM^ Green Master Mix	ThermoFisher, Waltham, MA, USA
430904	ELISA MAX^TM^ Deluxe Set Mouse TNF-α	Biolegend, San Diego, CA, USA
431304	ELISA MAX^TM^ Deluxe Set Mouse IL-6	Biolegend
432604	ELISA MAX^TM^ Deluxe Set Mouse IL-1β	Biolegend
HY-P80725	iNOS	MCE, Monmouth Junction, NJ, USA
HY-P80501	Iba1	MCE
14358S	Toll-like Receptor 4	CST, Danvers, MA, USA
5206T	TAK-1	CST
9339S	Phospho-TAK1	CST
61294S	IKKα	CST
2697S	Phospho-IKKα/β	CST
8242S	NF-κB p65	CST
3033S	Phospho-NF-κB p65	CST
4812S	IκBα	CST
2859S	Phospho-IκBα	CST
97166S	GAPDH	CST
13435S	Lamin B1	CST
P0131	Anti-fluorescence quenching mounting solution (containing DAPI)	Beyotime
15101S	NLRP3	CST
67824T	ASC/TMS1	CST
HY-P81232	Caspase-1	MCE
HY-P80622	Cleaved-Caspase 1	MCE
12703T	IL-1β	CST
63124T	Cleaved-IL-1β	CST
HY-P80241	NeuN	MCE
AC12L054	EZ 488 TUNEL apoptosis detection kit	Life-iLab, Shanghai, China
C1062L	Annexin V-FITC apoptosis detection kit	Beyotime
3498T	Bcl-2	CST
14796T	Bax	CST
9662T	Caspase 3	CST
9661T	Cleaved caspase 3	CST
BL767A	DAF-FM DA	Biosharp, Hefei, China

**Table 2:** Primers for RT-PCR of cell samples.

Name of Primers	Sequence
TNF-α Forward	5′-CCTATGTCTCAGCCTCTTCT-3′
TNF-α Reverse	5′-CCTGGTATGAGATAGCAAAT-3′
IL-6 Forward	5′-CCACTTCACAAGTCGGAGGCTT-3′
IL-6 Reverse	5′-CCAGCTTATCTGTTAGGAGA-3′
iNOS Forward	5′-GTTCTCAGCCCAACAATACAAGA-3′
iNOS Reverse	5′-GTGGACGGGTCGATGTCAC-3′
IL-1β Forward	5′-ACTCATTGTGGCTGTGGAGA-3′
IL-1β Reverse	5′-ACTCATTGTGGCTGTGGAGA-3′
β-actin Forward	5′-ATCCTGA AAGACCTCTATGC-3′
β-actin Reverse	5′-AACGCAGCTCAGTAACAGTC-3′

**Table 3:** Primers for RT-PCR of zebrafish samples.

Name of Primers	Sequence
iNOS Forward	5′-GGAGATGCAAGGTCAGCTTC-3′
iNOS Reverse	5′-GGCAAAGCTCAGTGACTTCC-3′
IL-1β Forward	5′-CATTTGCAGGCCGTCACA-3′
IL-1β Reverse	5′-GGACATGCTGAAGCGCACTT-3′

## Data Availability

All data associated with this study are presented in this manuscript.

## Supplementary Materials

The following supporting information can be downloaded at: https://www.mdpi.com/article/10.3390/ijms27146440/s1.
