# Supplementary material for: Glycine tabacina (Labill.) Benth. Ethanol Extract Attenuates LPS-Induced Neuroinflammation and Behavioral Deficits by Modulating TLR4/NF-κB/NLRP3 Signaling Pathway
Source: Int J Mol Sci. 2026 Jul 20;27(14):6440. doi: 10.3390/ijms27146440 (PMC13411664; doi:10.3390/ijms27146440)
Supplement: Supplementary file 1 [file ijms-27-06440-s001.zip › ijms-4416389-supplementary.pdf]

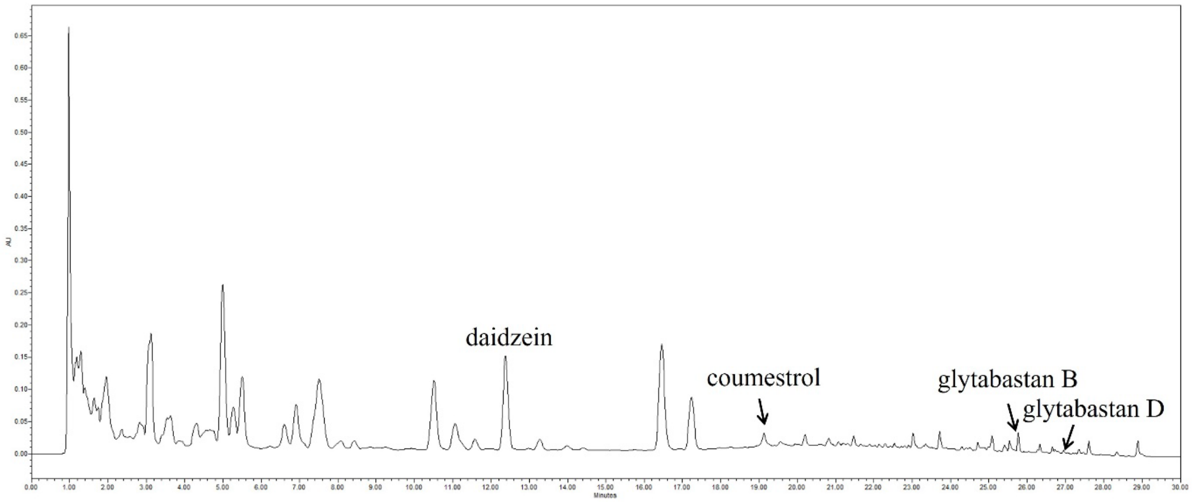

Fig. S1. Representative UPLC fingerprint chromatogram of GTE at 255nm.

Table S1 Content of Compounds in GTE

Compound	Content (%, w/w)
glytabastan B	0.0171 ± 0.0105
glytabastan D	0.0020 ± 0.0011
coumestrol	0.0266 ± 0.0054
daidzein	0.6685 ± 0.2554
